# Erebosis of Neurons May Exist in the Brain with Alzheimer’s Disease

**DOI:** 10.3390/cells14191546

**Published:** 2025-10-03

**Authors:** Jun Li, Zhiyi Zuo

**Affiliations:** Department of Anesthesiology, University of Virginia, Charlottesville, VA 22908, USA

**Keywords:** Angiotensin-converting enzyme 2, Alzheimer’s disease, erebosis, neuron, phospho-tau

## Abstract

**Highlights:**

**What are the main findings?**
  - Erebosis may be present in the neurons of mammalian brains and may be increased with aging and Alzheimer’s disease neuropathology.
  - Phospho-tau may be a stimulus to induce erebosis in the brain.
**What is the implication of the main findings?**
  - Reducing erebosis may be a strategy to decrease cell loss/death in the brain of patients with Alzheimer’s disease.
  - Erebosis may be a form of phospho-tau-induced neurotoxicity.

**Abstract:**

Erebosis is a newly described form of cell death but has been reported only in the gut enterocytes of *Drosophila*, a group of fast turnover cells. Angiotensin-converting enzyme 2 (ACE2) accumulation in cells is a biomarker for erebotic cells. Brain cell loss is a characteristic of patients with Alzheimer’s disease (AD), the leading neurodegenerative disease. The objectives of this study are to determine whether there is erebosis in the mammalian brain. Here we show that there is more ACE2 staining in the hippocampus of old wild-type (C57BL/6J) male mice, female mice with AD neuropathology (3xTg-AD mice), and human AD sufferers. Some ACE2 positive cells have fragmented or small nuclei, lose NeuN staining and are positive for TUNEL staining, indicators for cell injury/dying. ACE2 positive cells are neurons in the hippocampus and are often positive for phospho-tau in the mice with AD neuropathology. Phospho-tau injected into the hippocampus of wild-type young adult mice increases its ACE2 expression. Some ACE2 staining is extracellular. Our results suggest that erebosis may exist in the mammalian brain and may be increased with aging and AD neuropathology. This form of death may occur in the long-lasting cells like neurons and can be activated by phospho-tau in the brain. Our findings highlight the therapeutic potential of regulating erebosis for attenuating brain aging and AD neuropathology.

## 1. Introduction

Alzheimer’s disease (AD) is a major neurodegenerative disease affecting a large number of elderly patients and a leading cause of mortality and morbidity in the world [1]. The two neuropathological hallmarks in patients with AD are amyloid plagues formed by amyloid peptides and intracellular tangles consisting of hyperphosphorylated tau [2,3,4]. Brain cell loss is a character of AD brain [5], and is considered as a basis for the cognitive impairment of patients with AD [6]. Many forms of cell death have been reported in the AD brain [7]. For example, apoptosis is increased in the brain of patients with AD [8]. Erebosis is a new form of cell death first reported in 2023 in the gut enterocytes of *Drosophila* [9]. However, it is not known whether erebosis exists in mammalians and organs other than the gut, and its biological implications are unknown [9,10].

Erebosis was found in the enterocytes, cells with fast turnover. Erebotic cells lose adhesion with the neighboring cells, nuclei, and organelles, and have the accumulation of angiotensin-converting enzyme 2 (ACE2). Such accumulation of ACE2 may be due to the uptake of the extracellular ACE2 into the erebotic cells. Erebosis is considered to play a role in maintaining gut barrier integrity via the natural shedding of the enterocytes [9]. This study is designed to determine whether erebosis exists in the mammalian brains and whether the degree of erebosis is changed with aging or in the presence of AD neuropathology. To achieve these goals, young and old wild-type mice, mice with or without AD neuropathology, and human brain sections with or without AD neuropathology were used in the study.

## 2. Methods and Materials

### 2.1. Animals

Eight-week-old C57BL/6J male mice were purchased from Charles River (Wilmington, MA, USA), and 22-month-old male C57BL/6J mice were from the National Institutes of Health (Bethesda, MD, USA). A pair of 3xTg-AD mice [B6;129-Tg (APPSwe, TauP301L) 1Lfa Psen1^tm1Mpm^/MmJax] and a pair of AD-control mice (B6129SF2/J) from Jackson Laboratory (stock number: 034830 for 3xTg-AD mice, stock number: 101046 for AD control mice, Bar Harbor, ME, USA) were bred in our vivarium to produce the offspring mice. Two- and 14-month-old female 3xTg-AD mice, along with the same age and gender AD control mice, were used in this study. All animals were maintained in the vivarium under pathogen-free conditions (23 ± 2 °C; 12 h light/dark cycle) with free access to food and water and 3 to 5 mice were housed per cage before and during the experiments. All experimental procedures were approved by the Institutional Animal Care and Use Committee of the University of Virginia (Charlottesville, VA, USA) with the protocol number 3114 on 18 May 2022. All animal experiments were performed in accordance with the National Institutes of Health Guide for the Care and Use of Laboratory Animals (NIH publications number 80-23) revised in 2011.

Three sets of experiments were performed. The first one compared 8-week-old C57BL/6J male mice to 22-month-old male C57BL/6J mice. The second one compared 2-month-old female 3xTg-AD mice to 14-month-old female 3xTg-AD mice. The third experiments compared 8-week-old C57BL/6J male mice that received or did not receive an injection of Tau-441 K18 (P301L) fibrils into the hippocampus. Randomization was not needed in experiments 1 and 2. Randomization per computer-generated assignment of each mouse was performed for experiment 3. The experimental unit is the individual animal. A total of 48 mice were used in these studies. The brains of these mice were used for immunostaining and cell death staining. The quantification of the staining was performed in a blinded fashion. Dr. Jun Li knew the group assignment during the allocation of the animals and the injection of the Tau fibrils but then was blinded after the brain harvesting.

### 2.2. Human Brain Sections

Human brain tissue sections containing the hippocampus were provided by the University of Virginia Biorepository and Tissue Research Facility. These sections were 4 µm thick, formalin-fixed, and paraffin-embedded sections from autopsy tissues. Diagnosis of AD and normal brains was made by neuropathologists before our request for the sections. Randomization was not needed for these brain sections. These human brain sections were cut from previously stored brain blocks and did not contain any identifiable information. Thus, approval for using these brain sections from the Institutional Review Board was not needed. The experimental unit is the individual subject. Brain sections of a total of 11 subjects were used in this study. The sections were used for immunostaining. The quantification of the staining was performed in a blinded fashion. Dr. Zhiyi Zuo knew the diagnosis of the subjects and then gave the information to Dr. Jun Li after the quantification of the staining was completed.

### 2.3. Tau Preformed Fibrils

Human Tau-441 K18 (P301L) pre-formed fibril protein was purchased from Acro Biosystems (Newark, DE, USA) (catalog number: TAU-H5113). Aliquots of tau preformed fibrils were stored at −80 °C and were sonicated with 60 brief pulses before the stereotaxic injection.

### 2.4. Stereotaxic Surgery

Eight-week-old C57BL/6J male mice that were acclimated for at least 3 days in our vivarium were anesthetized with 100 mg/kg ketamine and 5 mg/kg xylazine, and were briefly head-restrained. Bilateral injections into the hippocampus were performed as we have described with the following stereotaxic coordinates: −2.5 mm from Bregma, +2.0 mm lateral from midline, and −1.8 mm in-depth. After injection, the needle was kept in place for an additional 5 min. Animals were kept on a heating pad throughout the surgical procedure and returned to their home cages after a 2 h post-surgery recovery and monitoring. A total volume of 5 μL solution containing 1.0 μg/μL Tau-441 K18 (P301L) fibrils was injected, and the same volume of phosphate-buffered saline (PBS, solution for the tau preformed fibrils) was injected into the sham mice. Animals were monitored for pain, suffering, and distress as described in our animal use and care protocol. The brain was collected 4 weeks later for immunofluorescent staining.

### 2.5. Brain Harvesting

Mice were euthanized by isoflurane overdose. Mice were then perfused transcardially with normal saline. Brain was removed for further process.

### 2.6. Immunohistochemical Staining

The immunofluorescent labeling was performed as we have described before [11,12]. Briefly, the brains of the mice used in the study were harvested and post-fixed in 4% paraformaldehyde in 0.1 M PBS at 4 °C for 24 h, and then incubated in 30% PBS-sucrose solution overnight at 4 °C before being frozen in the cutting compound. Coronal 20-μm sections containing the hippocampus from Bregma −2 to −4 mm were cut using a cryostat (SM2010R, Leica Biosystems, Deer Park, MD, USA) and mounted on the microscope slides. After being washed in Tris-buffered saline (TBS) at room temperature, sections were blocked in 10% donkey serum plus 1% bovine serum albumin in TBS containing 0.25% Triton-X 100 for 2 h at room temperature and then incubated at 4 °C overnight with the primary antibodies: anti-ACE2 antibody (1:500, rabbit, Invitrogen, catalog number: MA5-32307 or 1:500, rat, R&D System, catalog number: MAB3437, Waltham, MA, USA), anti-phospho-tau (AT8) antibody (1:500, mouse, Invitrogen, catalog number: MN1020), anti-neuronal nuclei (NeuN) antibody (1:500, mouse, Abcam, catalog number: ab104224, Cambridge, UK), anti-cleaved-caspased-3 antibody (1:500, rabbit, Cell Signaling, catalog number: 9664, Lane Danvers, MA, USA), anti-microtubule-associated protein 1A/1B light chain 3B (LC3B) antibody (1:500, rabbit, Abcam, catalog number: ab192890), anti-ionized calcium binding adaptor molecule 1 (Iba1) antibody (1:500, rabbit, FUJIFILM Wako Pure Chemical Corporation, Osaka, Japan, catalog number: 01-19741), or anti-glial fibrillary acidic protein (GFAP) antibody (1:500, goat, Abcam, catalog number: ab53554). On the second day, the sections were rinsed in TBS three times, then incubated with the fluorophore-conjugated secondary antibodies for 2 h at room temperature in the dark. After being washed in TBS three times, sections were mounted with Prolong^TM^ Diamond Antifade Mountant (Invitrogen, Catalog number: P36970) mounting medium.

Immunofluorescent staining of human brain sections was performed briefly as follows. Antigen retrieval was performed by incubating sections with sodium citrate buffer containing 10 mM sodium citrate containing 0.05% Tween 20 (pH 6.0) at 95–100 °C. After being washed in TBS, sections were blocked in 10% donkey serum plus 1% bovine serum albumin in TBS containing 0.25% Triton X-100 for 2 h at room temperature and then incubated at 4 °C overnight with the following primary antibodies: anti-ACE2 antibody (1:500, rabbit, Invitrogen, catalog number: MA5-32307 or 1:500, rat, R&D System, catalog number: MAB3437), anti-phospho-tau (AT8) antibody (1:500, mouse, Invitrogen, catalog number: MN1020), anti-cleaved-caspased-3 antibody (1:500, rabbit, Cell Signaling, catalog number: 9664), or anti-LC3B antibody (1:500, rabbit, Abcam, catalog number: ab192890). On the second day, the sections were rinsed in TBS three times and then incubated with the fluorophore-conjugated secondary antibodies for 2 h at room temperature in the dark. After being washed in TBS three times, sections were mounted with Prolong^TM^ Diamond Antifade Mountant mounting medium.

### 2.7. Terminal Deoxynucleotidyl Transferase dUTP Nick End Labeling (TUNEL) Assay

The TUNEL assay was performed using the Click-iT^TM^ Plus TUNEL detection kit (Invitrogen, catalog number: C10618) according to the manufacturer’s instructions. Briefly, after permeabilization with proteinase K solution and PBS washes, the mouse brain sections were incubated with TdT reaction buffer for 10 min at 37 °C, with TdT reaction mixture for 60 min at 37 °C, and then with Click-iT^TM^ Plus TUNEL reaction cocktail for 30 min at 37 °C protected from light. Samples were then stained with other primary antibodies as described in the immunofluorescent labeling process.

### 2.8. Quantification of Immunohistochemistry

Staining images were acquired with a Stellaris 8 confocal microscopy system (Leica). To quantify the staining of ACE2, the CA1, CA2, and CA3 regions covered by four non-overlapping fields from each of the four sequential sections of one mouse were imaged (these 4 fields covered almost the whole CA2 and CA3 regions and randomly selected 4 fields were imaged in the CA1 regions). The mean intensity per image above a predetermined threshold level was considered a positively stained area for an interested marker and quantified using Image J version 1.60. Similarly, 4 non-overlapping fields were taken in the hippocampus of each human brain section (two sections per individual). The immunofluorescence intensity was measured by using Image J. The number of cells with positive staining for both ACE2 and AT8 was counted in two randomly selected and independent microscopic fields per section with four sections in the CA1, CA2, and CA3 regions of each mouse. The total number of ACE2 or AT8 positive cells in the same microscopic fields was counted to calculate the proportion of ACE2 and AT8 positive cells among all ACE2- or AT8 positive cells.

### 2.9. Statistical Analysis

For the animal studies, we aimed to have a sample size of 6 mice per group. For human brain section studies, the sample size was 5 to 6 per group depending on the maximal number of subjects that met the requirements for this study (clearly diagnosed by a neuropathologist to be normal brain or brain with AD neuropathology and there was hippocampus in the stored brain blocks) from the University of Virginia Biorepository and Tissue Research Facility. These sample sizes were not determined by power analysis but were based on our previous experience. Data of all animals in the studies were included for analysis. We did not set up any exclusion criteria prior to the experiments.

Parametric results in normal distribution are presented as mean ± S.D. (n ≥ 5). Non-parametric data, parametric data in non-normal distribution or data with an n = 4 are presented in box plot. The data were analyzed by unpaired t-test (parametric data in normal distribution) or rank sum test (parametric data in non-normal distribution). Normality of the data was tested by Shapiro–Wilk test. Differences were considered significant at *p* < 0.05 based on two-tailed hypothesis testing. All statistical analyses were performed with GraphPad Prism 8.0.

## 3. Results

### 3.1. Erebosis May Exist in the Mouse Hippocampus and May Be Increased with Aging and AD Neuropathology

**Figure 1:**
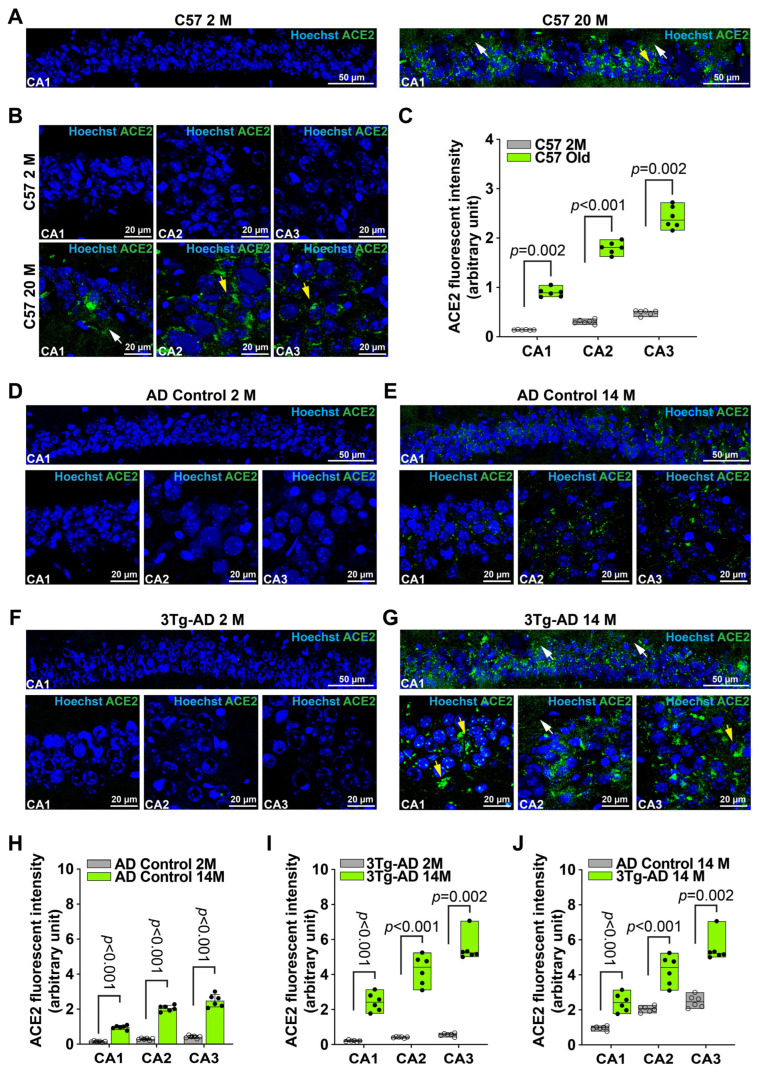
ACE2 in the hippocampus is increased with aging and in the brain with AD neuropathology in mice. (**A**), Immunofluorescence images of CA1 regions of C57BL/6J mice in low magnifications. Scale bar = 50 µm. (**B**), Immunofluorescence images of CA1, CA2, and CA3 regions of C57BL/6J mice in high magnifications. Scale bar = 20 µm. (**C**), Quantification results of ACE2 expression in the hippocampus of C57BL/6J mice. Results are in box plot (n = 6 mice, rank sum test). (**D**,**E**), Immunofluorescence images of the hippocampus of control mice. Scale bar = 50 µm in the upper panels and = 20 µm in the lower panels. (**F**,**G**), Immunofluorescence images of hippocampus of 3xTg-AD mice. Scale bar = 50 µm in the upper panels and = 20 µm in the lower panels. (**H**–**J**), Quantification results of ACE2 expression in the hippocampus of 3xTg-AD mice and their control mice. Results are mean ± SD or in box plot (n = 6 mice, *t* test or rank sum test). Yellow arrows in the images indicate cells that are positive for ACE2 but have fragmented nuclei or small nuclei. White arrows in the images indicate extracellular ACE2 staining.

To determine whether there was erebosis in the brain, we performed immunofluorescence staining of ACE2. We focused on the hippocampus because the hippocampus is critical for learning and memory and has significant pathology in patients with AD [6,13,14]. As shown in Figure 1, ACE2 staining was increased in the CA1, CA2, and CA3 of 20-month-old male C57BL/6J mice. Consistent with the nature that ACE2 is a secreted protein [15] and the finding of a previous study [9], some of the ACE2 staining appeared extracellular. Some cells with positive staining for ACE2 had fragmented nuclei. However, very little or almost no ACE2 staining existed in the hippocampus of 2-month-old male C57BL/6J mice (Figure 1A–C and Figure S1). These results suggest that erebosis may exist in the hippocampus of old C57BL/6J mice.

The 3xTg-AD mice are an AD mouse model that has 3 human mutant genes for AD [16,17,18]. These mice started to have amyloid plagues and hyperphosphorylated tau at an age of 6 months and these pathological changes in the hippocampus are significant after the age of 12 months in female mice [17,18]. Since female 3xTg-AD mice have more consistent neuropathology than male mice [19], we used female 3xTg-AD mice and control mice in this study. Similarly to the findings in C57BL/6J mice, 2-month-old 3xTg-AD mice and their age-matched control mice (B6129SF2/J) had little ACE2 staining in the hippocampus. The ACE2 staining was increased in the 14-month-old 3xTg-AD mice and controls. Importantly, the ACE2 staining was higher in the 14-month-old 3xTg-AD mice than age-matched control mice (Figure 1D–J and Figure S2). These results suggest that erebosis may be increased in the hippocampus with AD neuropathological changes in mice.

### 3.2. Phospho-Tau May Be an Inducer for Erebosis and Erebosis May Occur in the Neurons of Hippocampus

**Figure 2:**
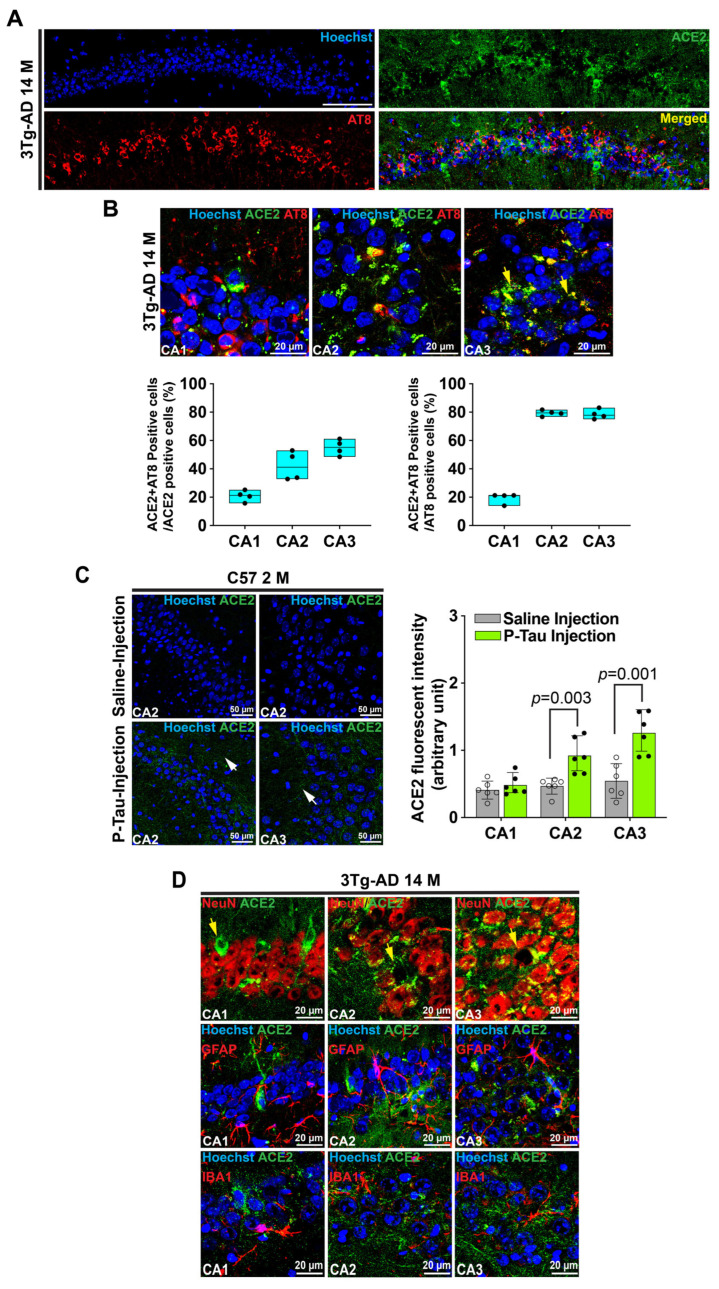
Phospho-tau contributes to the increase in ACE2, and ACE2 positive cells are neurons. (**A**), Immunofluorescence staining of ACE2 and hyperphospho-tau at ser 202 and thr 205 as detected by the AT8 antibody in CA1 of 3xTg-AD mice in low magnifications. Scale bar = 100 µm. (**B**), Upper panel: immunofluorescence images of CA1, CA2, and CA3 regions of 3xTg-AD mice in high magnifications. Scale bar = 20 µm. Lower panel: quantification of cells positive for both ACE2 and hyperphospho-tau in cells that are positive for ACE2 or hyperphospho-tau in the hippocampus of 3xTg-AD mice. Results are in box plot (n = 4 mice). (**C**), Immunofluorescence images of ACE2 in CA2 and CA3 of C57Bl/6J mice (left panel, scale bar = 50 µm) and quantitative results of ACE2 (right panel, results are mean ± SD, n = 6 mice, *t*-test). (**D**), Co-staining of ACE2 and NeuN, GFAP and Iba1. Scale bar = 20 µm. Yellow arrows in the images indicate cells that are positive for ACE2 but have fragmented nuclei, small nuclei, or lack of nuclei. White arrows in the images indicate extracellular ACE2 staining.

Since ACE2 is accumulated intracellularly and accumulation of hyperphosphorylated tau inside cells is a feature of the AD brain [2,3,4], we determined whether phospho-tau contributes to the accumulation of ACE2 in the cells. About 21%, 42%, and 55% cells in the CA1, CA2, and CA3, respectively, were positive for the staining of both ACE2 and hyperphospho-tau at ser 202 and thr 205 as detected by the AT8 antibody in the cells that were positive for ACE2. About 19%, 79%, and 78% cells in the CA1, CA2, and CA3, respectively, were positive for staining of both ACE2 and hyperphospho-tau at ser 202 and thr 205 in the cells that were positive for hyperphospho-tau at ser 202 and thr 205 (Figure 2A,B and Figure S3). These findings suggest that hyperphospho-tau may facilitate the accumulation of ACE2 in the cells. Injecting phospho-tau but not saline to the hippocampus of 2-month-old male C57BL/6J mice increased the staining of ACE2 in the CA2 and CA3. However, some ACE2 staining appeared in the extracellular space (Figure 2C, Figures S4, and S5). These results provide direct evidence that phospho-tau stimulates the expression of ACE2 and that ACE2 is preferably accumulated in the cells with the accumulation of hyperphosphorylated tau.

The staining of ACE2 was co-localized with NeuN and was not co-localized with GFAP and Iba-1. For those cells without NeuN positive staining, ACE2 positive cells had a neuronal cell appearance (Figure 2D, Figures S6 and S7). These results suggest that ACE2 positive cells are neurons and may not be microglia and astrocytes and that some of the ACE2 cells may have lost NeuN proteins in their nuclei, indicating alternations or damages in the nuclei of these cells.

### 3.3. Accumulation of ACE2 May Be a Sign of Erebosis in the Brain

**Figure 3:**
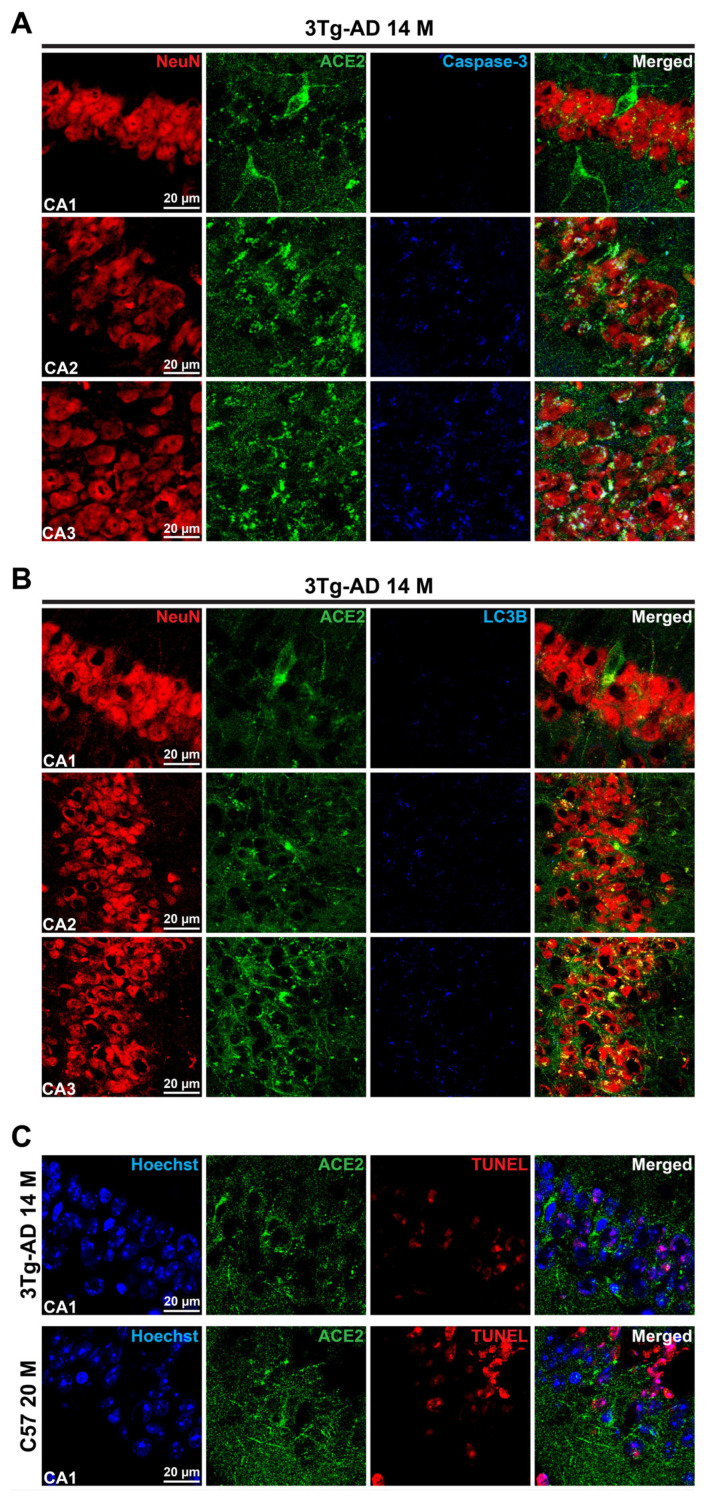
Co-staining of ACE2 with cleaved caspase 3, LC3B or TUNEL. (**A**), Co-staining images of ACE2 and cleaved caspase 3 of 3xTg-AD mice. Scale bar = 20 µm. (**B**), Co-staining images of ACE2 and LC3B of 3xTg-AD mice. Scale bar = 20 µm. (**C**), Co-staining images of ACE2 and TUNEL of 3xTg-AD mice and old C57BL/6J mice. Scale bar = 20 µm.

To determine whether ACE2 positive cells may be apoptotic cells, triple staining for NeuN, ACE2, and cleaved caspase 3 was performed. There was very little staining of cleaved caspase 3 in the CA1, although there were significant ACE2 positive cells. Some of the ACE2 staining in the CA2 and CA3 was co-localized with the cleaved caspase 3 staining (Figure 3A and Figure S8), suggesting that some of the ACE2 positive cells have activated caspase 3 expression. Similarly, there was ACE2 staining but no LC3B staining in the CA1. There was some LC3B staining in the CA2 and CA3. However, it did not appear that the LC3B staining existed in cells that were strongly positive for ACE2 staining in these brain regions (Figure 3B and Figure S9). These results suggest that ACE2 positive cells may not be autophagic cells.

**Figure 4:**
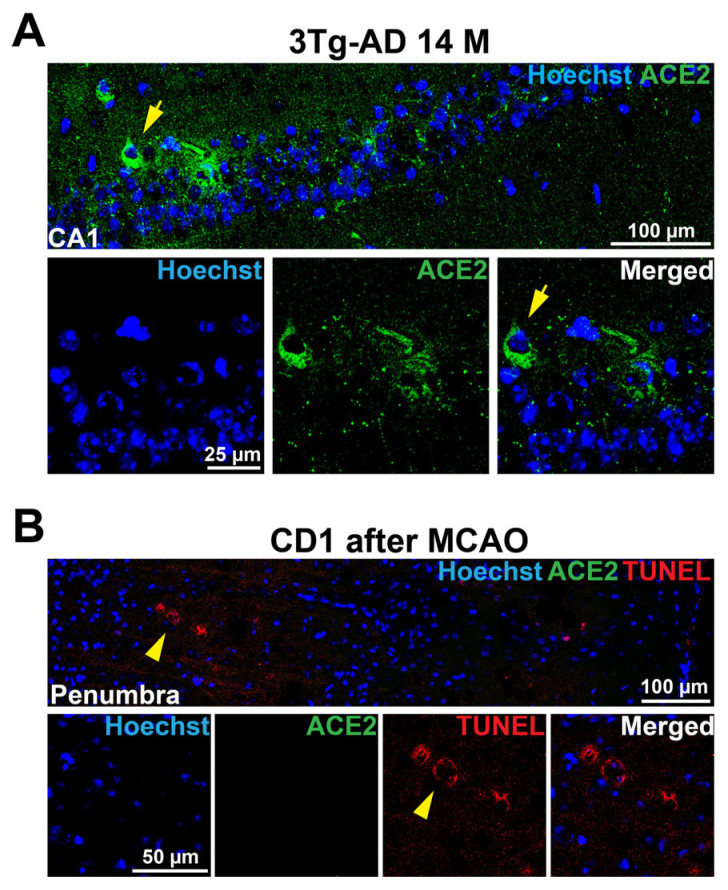
ACE2 staining may be specific for erebosis. (**A**), Immunofluorescence images of the CA1 region of 3xTg-AD mice. Scale bar = 100 µm in the upper panel and = 25 µm in the lower panels. (**B**), Immunofluorescence images of ischemic penumbra of CD-1 mice after middle cerebral arterial occlusion (MCAO). Scale bar = 100 µm in the upper panel and = 50 µm in the lower panels. Yellow arrows in panel (**A**) indicate cells that are positive for ACE2 staining. Yellow arrow heads in panel (**B**) indicate cells that are positive for TUNEL staining.

A cell that is TUNEL staining positive is considered an injured/dying cell [9,20]. There were TUNEL staining positive cells in the brain of C57BL/6J and 3xTg-AD mice. Some ACE2 staining positive cells were TUNEL staining positive (Figure 3C, Figures S10 and S11), suggesting that these cells are dying. ACE2 accumulation in cells is considered a sign of erebosis [9]. To determine whether ACE2 is accumulated in other forms of cell death, we used brain sections of 2-month-old CD-1 male mice that had a 90-mim episode of right middle cerebral arterial occlusion to create a focal brain ischemic region as in our previous study [21]. The brain was harvested 24 h after the arterial occlusion to cut the sections. Cells in the ischemic penumbral region, a region surrounding the infarct core, had various forms of cell death including necrosis and apoptosis [22]. As anticipated, there were TUNEL positive cells in the ischemic penumbra. However, there was no ACE2 staining in this region. On the other hand, brain sections of 14-month-old 3xTg-AD mice stained simultaneously with those brain sections of CD-1 mice were positive for ACE2 staining (Figure 4). The 14-month-old 3xTg-AD mouse sections were included as positive control for the staining process. These results suggest the specificity of ACE2 staining for erebosis.

### 3.4. Erebosis May Occur in the Hippocampus of the Human Brain

**Figure 5:**
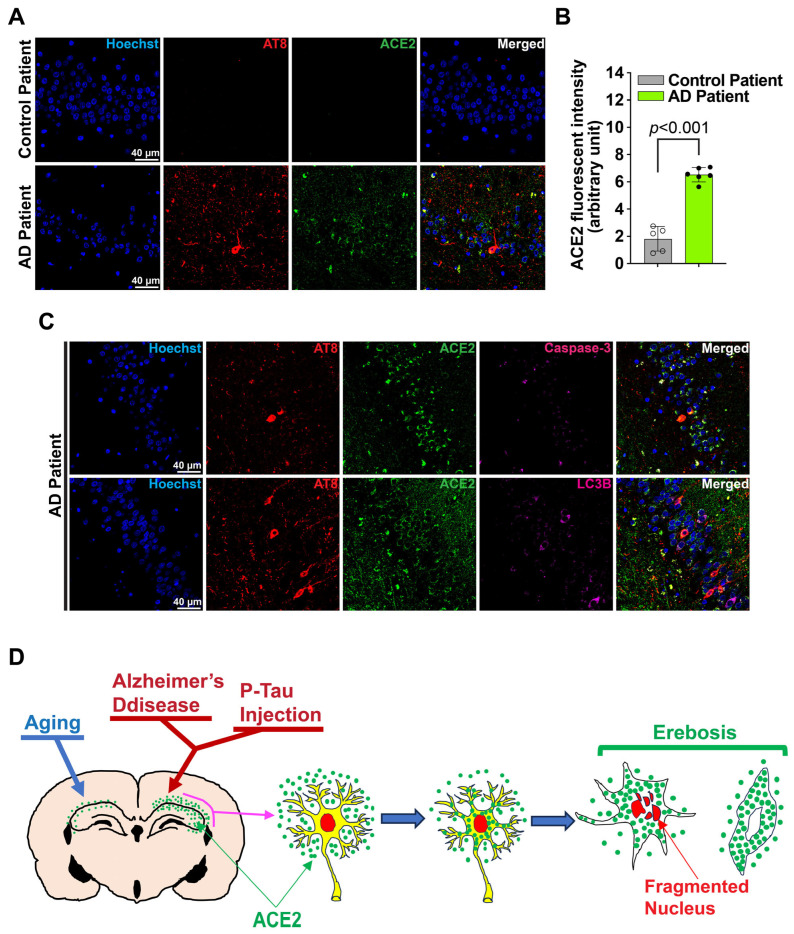
ACE2 is increased in the hippocampus with AD neuropathology. (**A**), Immunofluorescence images of the human hippocampus. Scale bar = 40 µm. (**B**), Quantification results of ACE2 expression in the human hippocampus. Results are mean ± SD (n = 5–6 individuals, *t* test). (**C**), Co-staining images of ACE2 and cleaved caspase 3 or LC3B in the human hippocampus. Scale bar = 40 µm. (**D**), Diagram of the findings from this study.

Sections of normal brains and brains with AD are from the Biorepository and Tissue Research Facility, University of Virginia. The sections were cut from the samples of autopsy. The diagnosis of AD brain or normal brains was made by neuropathologists. The age of the donors was 66.2 ± 11.9 and 82.2 ± 8.3 (*p* = 0.028) for normal brain and AD brain, respectively. There were 2 and 1 females in the normal and AD brain groups, respectively. Consistent with the findings in previous reports [23,24], human AD brain had increased ACE2 in the hippocampus. Some cells were positive for both ACE2 and hyperphospho-tau at ser 202 and thr 205 in these AD brains (Figure 5A,B and Figure S12). Similarly to the findings in the mice, some ACE2 positive cells were positive for the staining of cleaved caspase 3 and LC3B (Figure 5C and Figure S13). These results suggest that many ACE2 positive cells are not apoptotic or autophagic cells in the human brain with AD.

## 4. Discussion

We have identified cells that accumulate ACE2 in the hippocampus of old wild-type mice and mice with AD brain changes. Accumulation of ACE2 is a feature of erebosis, a novel cell death form that has only been described in the gut enterocytes of *Drosophila* [9]. Similarly to the description in the first report on erebosis [9], some of the ACE2 positive cells have smaller or fragmented nuclei and are positive to TUNEL staining, suggesting that these cells may be dying. Importantly, the ACE2 positive cells are all neurons. Some of these ACE2 positive cells lost NeuN staining, suggesting the injury of these cells. Together, our results suggest that erebosis occurs in the neurons of old wild-type mice and mice with AD neuropathology. This finding indicates that erebosis occurs not only in the fast turnover cells like enterocytes but also in the long-lasting cells like neurons. Our study also provides initial evidence that erebosis exists in mammalian cells including cells of the human brain with AD neuropathology.

We showed that ACE2 increased in the brain with AD neuropathology. The increase in ACE2 in the human AD brain has been reported previously in two studies [23,24], although a study with a much smaller sample size (n = 5) suggests that ACE2 decreased in some of the brain regions [25]. The reason for this difference is not clear. Differences in the used techniques and agents to detect ACE2 expression and ethical origin of the brain tissues may have contributed to the different findings. In this regard, three studies including ours that show increased ACE2 expression in the human AD brains are from the United States and Canada, and the small study is from China. Interestingly, our study showed that the injection of phospho-tau into the brain of wild-type mice increased ACE2 expression and that there was a large fraction of brain cells that were positive for both ACE2 and hyperphospho-tau in the mice with AD pathology. These results suggest that phospho-tau may be an inducer for the cells to overexpress and accumulate ACE2 in the cells. However, the association between phospho-tau and ACE2 in the CA1 was low compared with that in the CA2 and CA3. The reasons for this phenomenon are not clear. It has been shown that CA1 cells are more sensitive to the ischemic insult than CA3 cells [26,27]. However, it is easier to induce the death of CA3 neurons than that of CA1 neurons by limbic seizure [28]. One possible explanation for these different sensitivities to various insults is that cells in different hippocampal regions do not use the same mechanisms to increase intracellular Zn^2+^ [29]. Thus, it is possible that phospho-tau may not be the major stimulus to increase ACE2 in the CA1 compared with that in the CA2 and CA3. Future studies may identify the major stimulus to increase ACE2 in the CA1.

Our study has shown that the erebotic cells are neurons but not microglia or astrocytes in the brain of 3xTg-AD mice. This finding may be anticipated because neurons are more sensitive to insults to die. Neurons have a high metabolic rate and various cell surface receptors that can overexcite them [30,31]. These mechanisms may contribute to the findings that erebotic cells are neurons.

It has been shown that the classical renin–angiotensin system (RAS) axis is overactivated and the regulatory RAS pathways including ACE2 are decreased with aging [25,32]. However, the results supporting these findings are mostly from studies measuring the RAS axis activity in the peripheral tissues. One study measuring ACE2 by ELISA showed that ACE2 was decreased with aging in mice with AD neuropathology and their control mice [33]. Another study did not find any changes in ACE2 expression in the brain across the ages from 4 months to 18 months of the 3xTg-AD mice and their control mice by Western blotting [23]. Our study showed an increase in ACE2 in the brain of 14-month-old 3xTg-AD mice compared with 2-month-old 3xTg-AD mice by the immunofluorescence staining method. These results are consistent with the findings from human brains as discussed above. Our study also showed that the expression of ACE2 was increased in the old wild-type mice. The reason for the different findings on ACE2 expression among studies is unclear. One clear difference is that we focused on the hippocampus and the two previous studies used brain homogenates of the cerebral cortex or the whole brain hemispheres except for the cerebrum [23,33]. These previous studies may have diluted the increase in ACE2 in the hippocampus by other brain tissues. In addition, we used monoclonal antibody to increase the specificity of our findings.

Evidence has suggested that apoptosis is a form of cell death in the brain of AD [8]. ACE2 positive cells may not be apoptotic because inhibiting apoptosis does not affect the number of cells expressing ACE2 in the previous study [9]. Our study showed that many of the ACE2 positive cells are not positive for cleaved caspase 3, a biomarker for apoptosis [8,34]. For example, there were ACE2 positive cells in the CA1 but no cleaved caspase 3 staining was shown in this region. These results suggest that ACE2 positive cells may not be apoptotic. Some ACE2 positive cells appeared positive for cleaved caspase 3 staining. It is not known yet whether erebosis and apoptosis can co-exist in the same cell or there is a relationship between ACE2 accumulation and caspase 3 activation. Autophagy may be decreased in the brain with AD [35]. There was staining of LC3B, a marker for autophagy [11,12], in the human brain with AD or mouse brain with AD neuropathology. However, the majority of ACE2 positive cells were not LC3B positive, and LC3B positive staining was not in the cells that were strongly positive for ACE2. These results suggest that ACE2 positive cells may not be autophagic.

ACE2 is a secreted protein [15]. Erebotic enterocytes may not increase the expression of ACE2 inside the cells, but the accumulation of ACE2 in these cells may be by taking up ACE2 into cells in the *Drosophila* [9]. There was extracellular ACE2 in the mouse brain with AD neuropathology, and phospho-tau injected into the wild-type mouse brain induced a large amount of extracellular ACE2. However, not all ACE2 positive cells were positive for phospho-tau staining. Thus, a possible mechanism for the increased ACE2 cells in the brain of AD is that phospho-tau stimulates the expression of ACE2 in these cells. Some or all of the expressed ACE2 is secreted to extracellular space. Cells then uptake ACE2 to initiate the erebotic processes (Figure 5D). However, it is not known what mechanisms are used to uptake ACE2 into these cells.

Our findings may have significant implications. We have shown that erebosis may occur in the neurons of brains with AD neuropathology in mice and humans. This finding may suggest that reducing erebosis may be a therapeutic approach to reduce brain cell death in the AD brain. Also, it appears that erebosis is increased with aging. Thus, erebosis of brain cells may contribute to the natural aging process of the brain. Interventions to reduce erebosis may be an approach to modulate this aging process.

One significant limitation with erebosis research is that the molecular pathways for erebosis remain unclear. As a result, it is difficult to define specific biomarkers for erebosis at this stage. Although ACE2 is a known biomarker for erebosis, it is hard to determine whether ACE2 is a trigger or a consequence of erebosis in the context that there is no other biomarker for erebosis. Nevertheless, our study may serve as an initial step to suggest that erebosis may exist in the mammalian cells and to promote studies to determine the molecular mechanisms and biomarkers for erebosis.

## 5. Conclusions

In summary, we have shown that erebosis may exist in the mammalian cells and may be increased in the brain with AD neuropathology and in the aged brain. Erebosis may be limited to the neurons in these brains. These findings provide solid bases for significant biological implications of erebosis in brain aging and AD, and may stimulate additional studies on erebosis.

## Data Availability

The data are available upon reasonable request.

## Supplementary Materials

The following supporting information can be downloaded at: https://www.mdpi.com/article/10.3390/cells14191546/s1, Figure S1. ACE2 is increased with aging in wild-type mice. Immunofluorescence images of the CA2 and CA3 regions of C57BL/6J mice in low magnifications. Figure S2. ACE2 is increased with aging in mice with AD neuropathology. Figure S3. ACE2 is in cells with hyperphospho-tau. Figure S4. ACE2 expression in 2-month old male C57BL/6J mice after receiving saline injection into the hippocampus. Immunofluorescence images of CA1, CA2 and CA3. Scale bar = 200 µm. Figure S5. ACE2 expression in 2-month old C57BL/6J mice after receiving phospho-tau fibril injection into the hippocampus. Immunofluorescence images of CA1, CA2 and CA3. Scale bar = 200 µm. Figure S6. ACE2 staining is not co-localized with the staining of GFAP in 3xTg-AD mice. Figure S7. ACE2 staining is not co-localized with the staining of Iba1 in 3xTg-AD mice. Figure S8. ACE2 and cleaved caspase 3 staining in 3xTg-AD mice. Figure S9. ACE2 and LC3B staining in 3xTg-AD mice. Figure S10. ACE2 and TUNEL staining in 3xTg-AD mice. Figure S11. ACE2 and TUNEL staining in old C57BL/6J mice. Figure S12. ACE2 and phospho-tau staining in human hippocampus. Figure S13. ACE2, phospho-tau, cleaved caspase 3 and LC3B staining in human hippocampus.
