# Supplementary material for: Erebosis of Neurons May Exist in the Brain with Alzheimer’s Disease
Source: Cells. 2025 Oct 3;14(19):1546. doi: 10.3390/cells14191546 (PMC12524094; doi:10.3390/cells14191546)
Supplement: Supplementary file 1 [file cells-14-01546-s001.zip › cells-3883051-supplementary.pdf]

Supplemental figures and legends

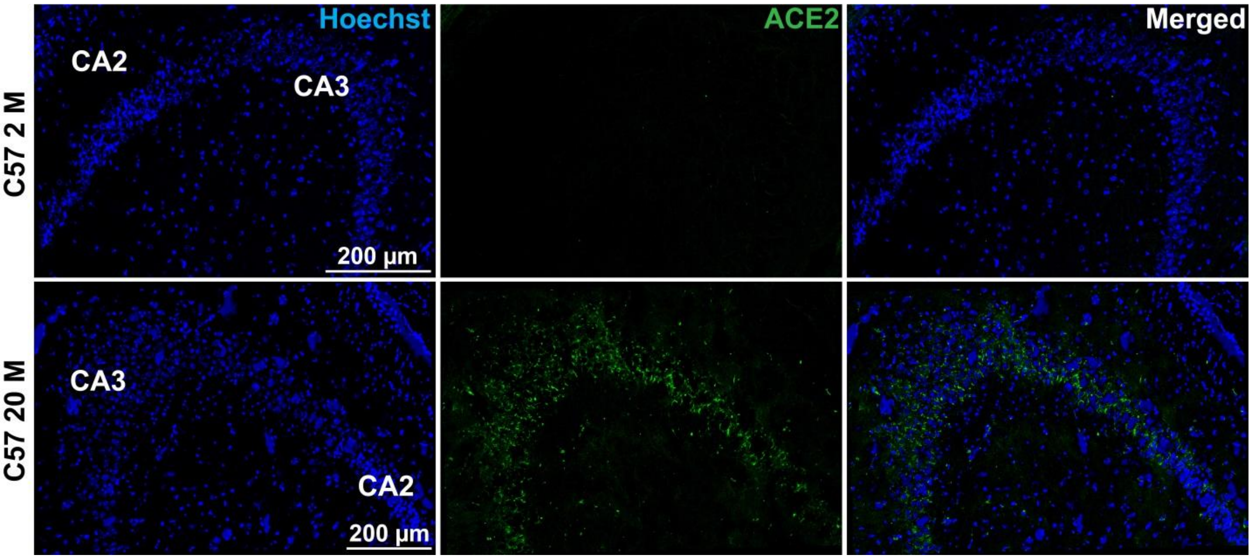

Fig. S1. ACE2 is increased with aging in wild-type mice. Immunofluorescence images of the CA2 and CA3 regions of C57BL/6J mice in low magnifications. Scale bar = 200 μm.

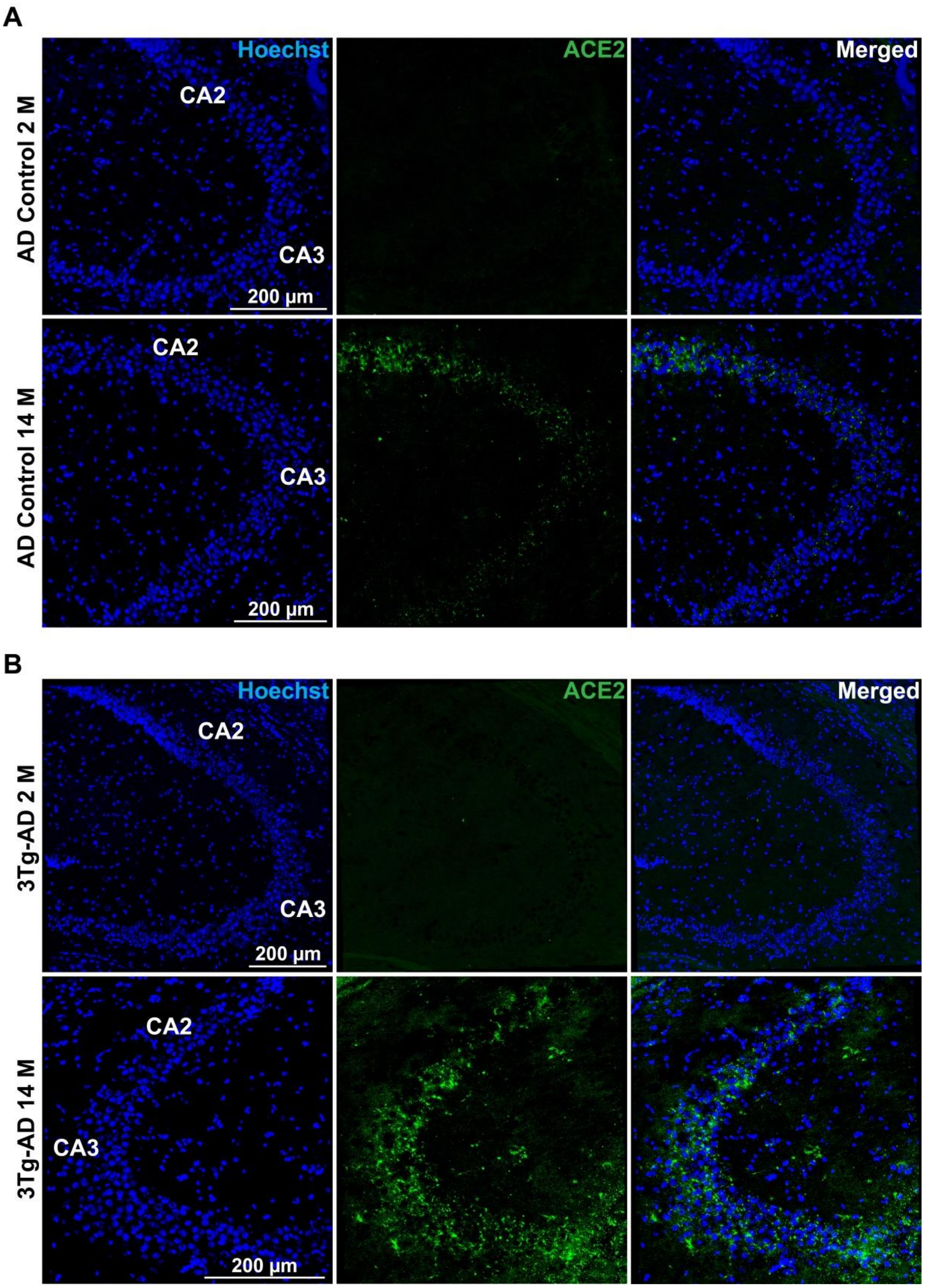

Fig. S2. ACE2 is increased with aging in mice with AD neuropathology. (**A**), Immunofluorescence images of CA2 and CA3 regions of control mice in low magnifications. Scale bar = 200 μm. (**B**), Immunofluorescence images of CA2 and CA3 regions of 3xTg-AD mice in low magnifications. Scale bar = 200 μm.

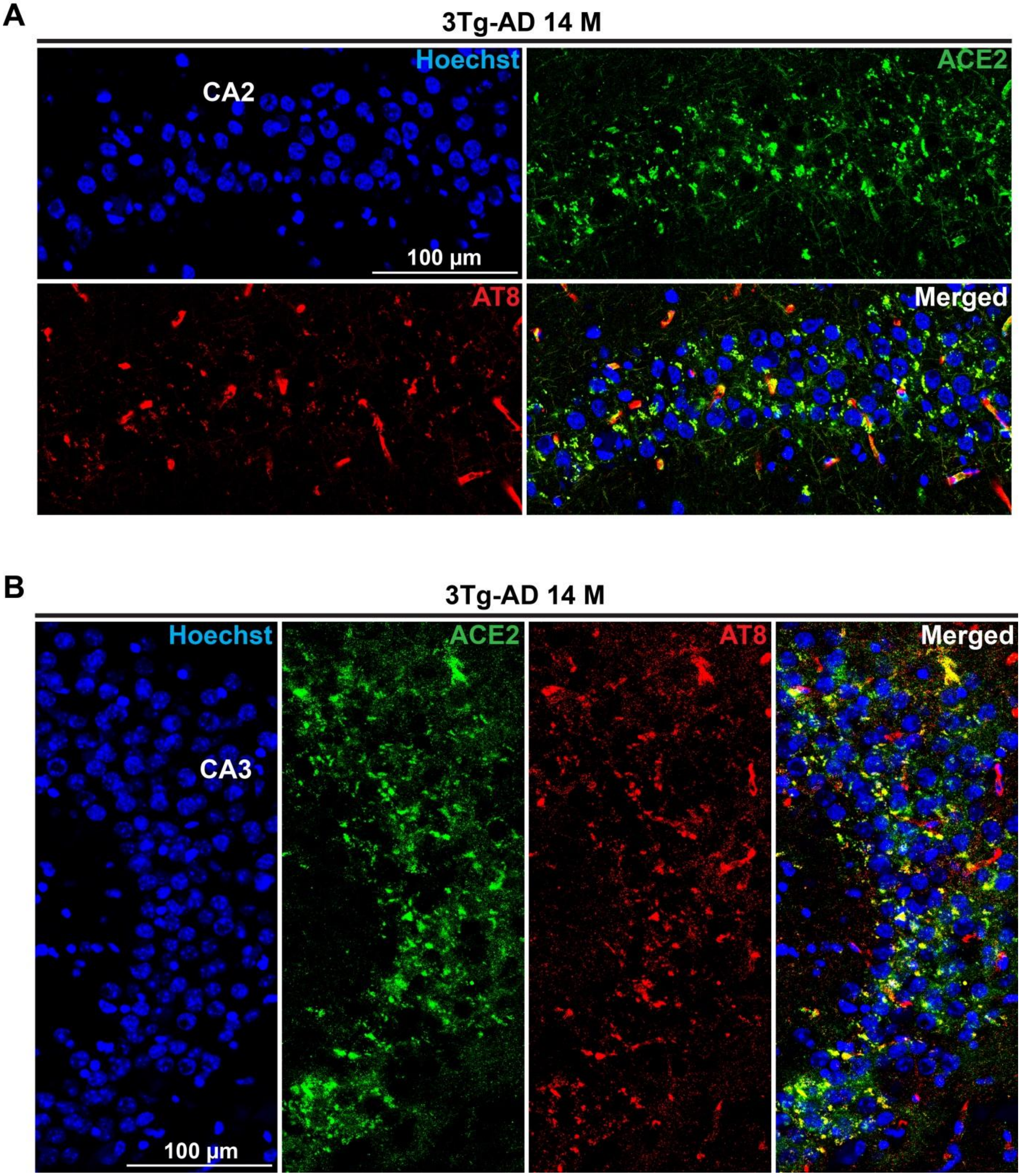

Fig. S3. ACE2 is in cells with hyperphospho-tau. (**A**), Immunofluorescence staining of ACE2 and hyperphospho-tau at ser 202 and thr 205 as detected by the AT8 antibody in the CA2 of 3xTg-AD mice in low magnifications. Scale bar = 100 µm. (**B**), Immunofluorescence staining of ACE2 and hyperphospho-tau at ser 202 and thr 205 as detected by the AT8 antibody in the CA3 of 3xTg-AD mice in low magnifications. Scale bar = 100 µm.

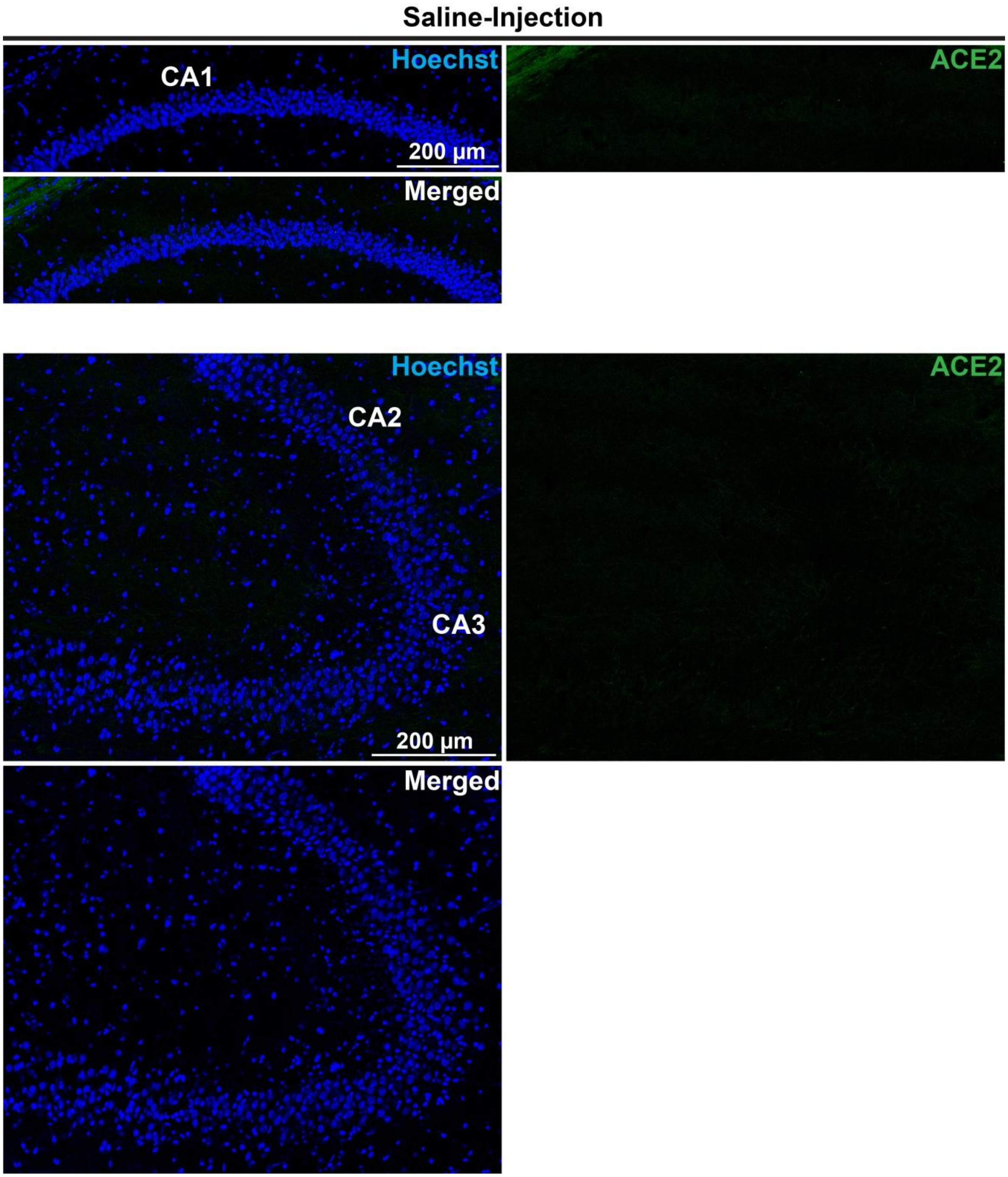

Fig. S4. ACE2 expression in 2-month old male C57BL/6J mice after receiving saline injection into the hippocampus. Immunofluorescence images of CA1, CA2 and CA3. Scale bar = 200 µm.

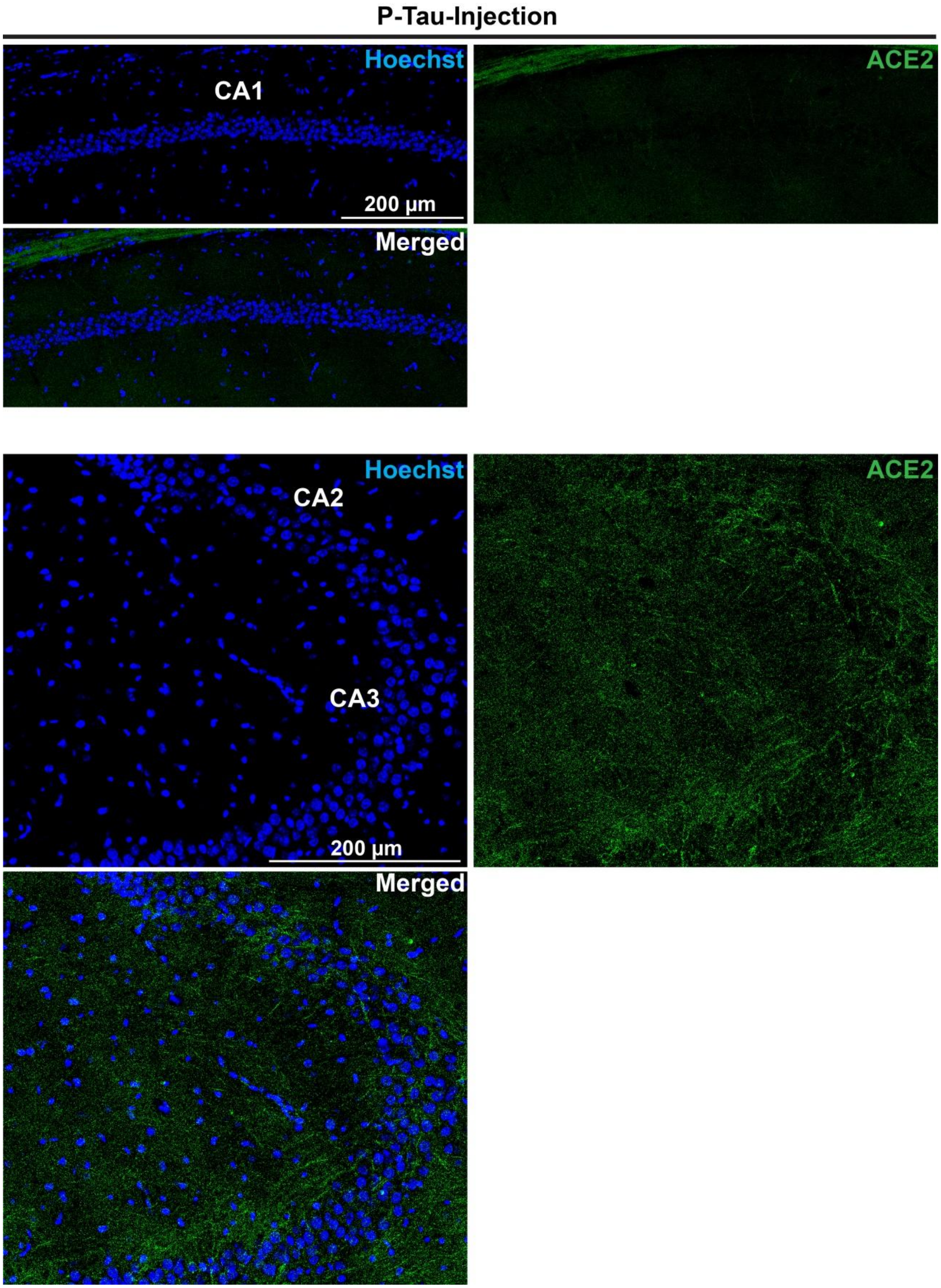

Fig. S5. ACE2 expression in 2-month old C57BL/6J mice after receiving phospho-tau fibril injection into the hippocampus. Immunofluorescence images of CA1, CA2 and CA3. Scale bar = 200 µm.

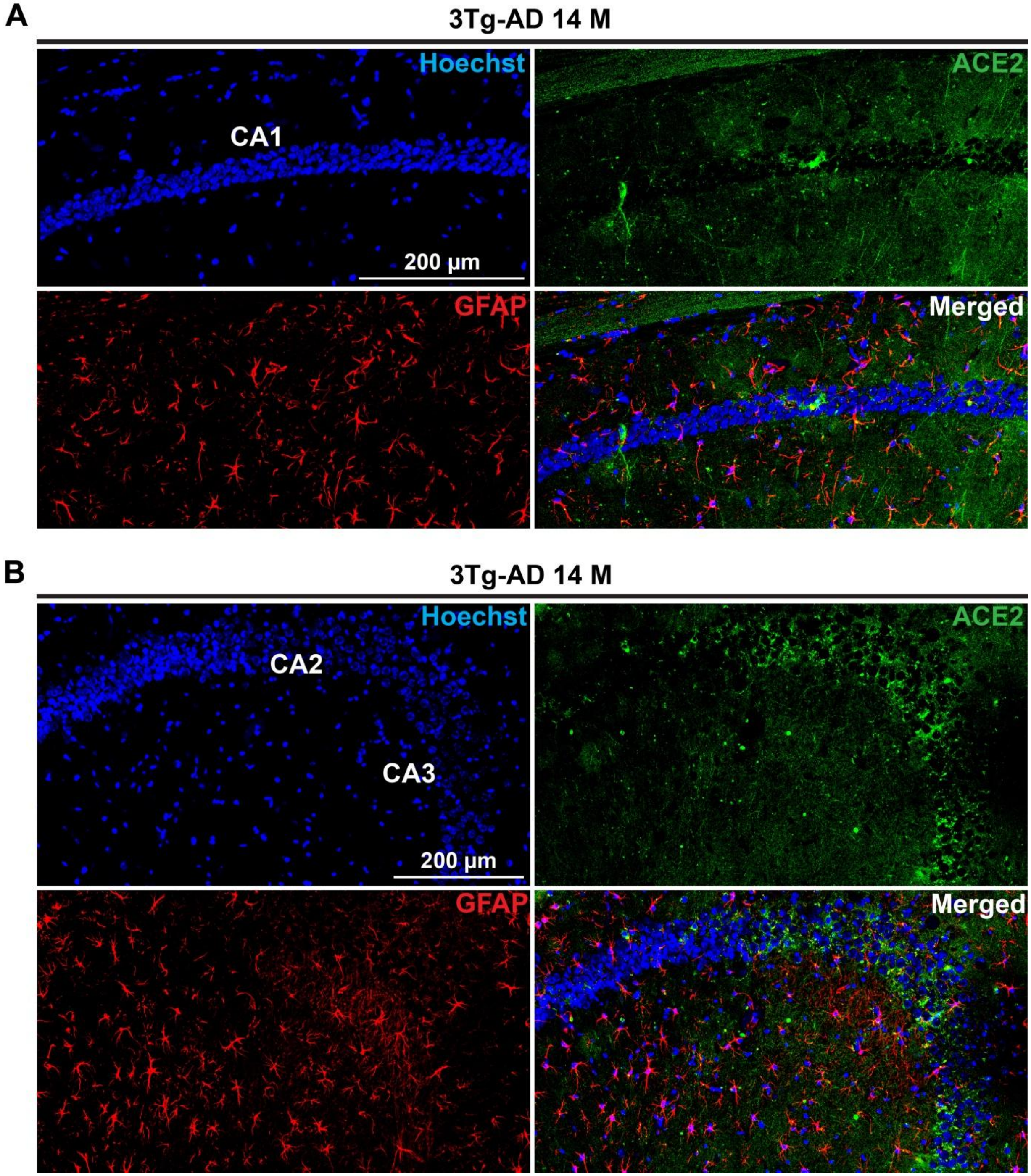

Fig. S6. ACE2 staining is not co-localized with the staining of GFAP in 3xTg-AD mice. (**A**), Immunofluorescence images of CA1. (**B**), Immunofluorescence images of the CA2 and CA3. Scale bar = 200 µm.

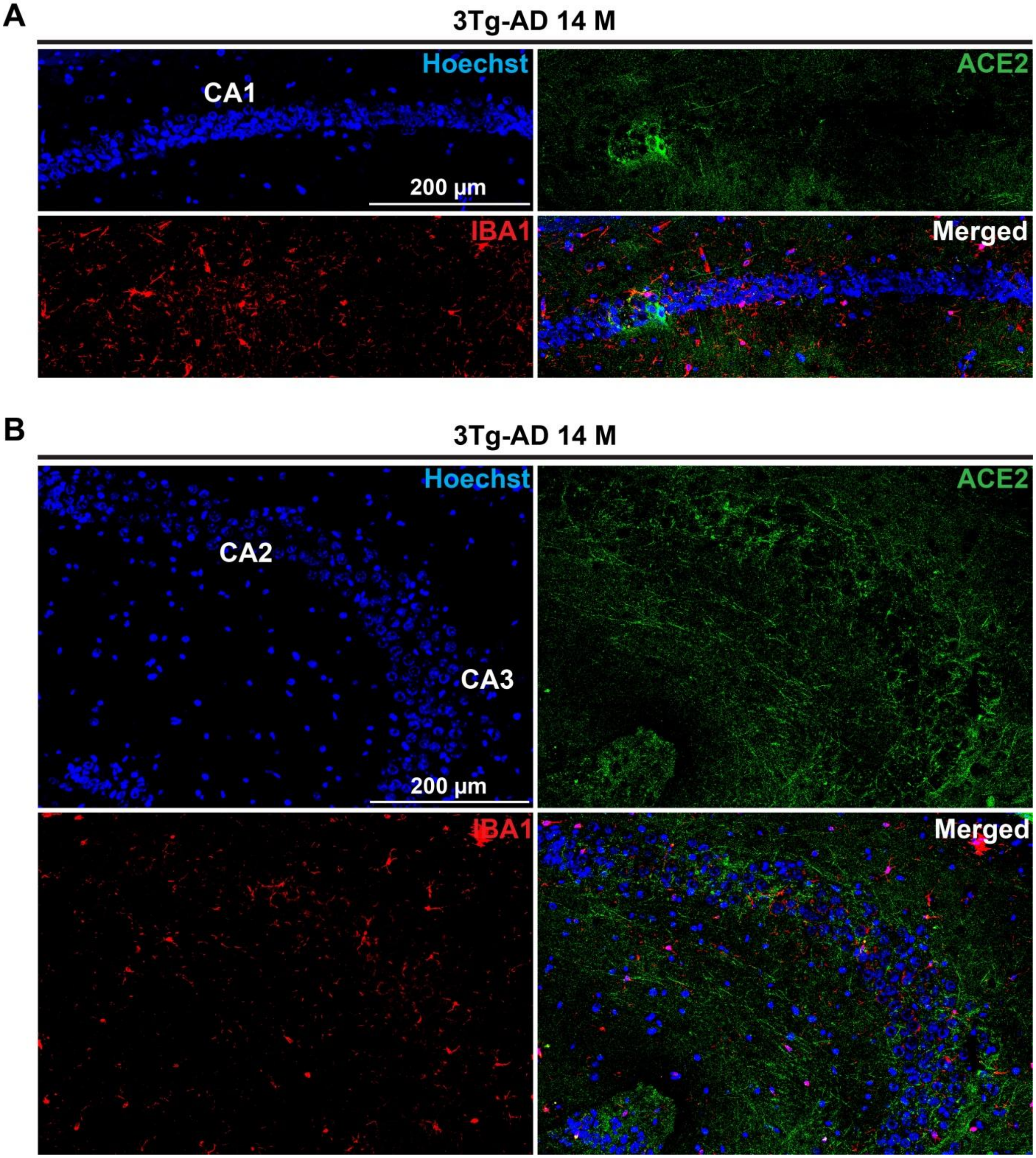

Fig. S7. ACE2 staining is not co-localized with the staining of Iba1 in 3xTg-AD mice. (**A**), Immunofluorescence images of CA1. (**B**), Immunofluorescence images of CA2 and CA3. Scale bar = 200 μm.

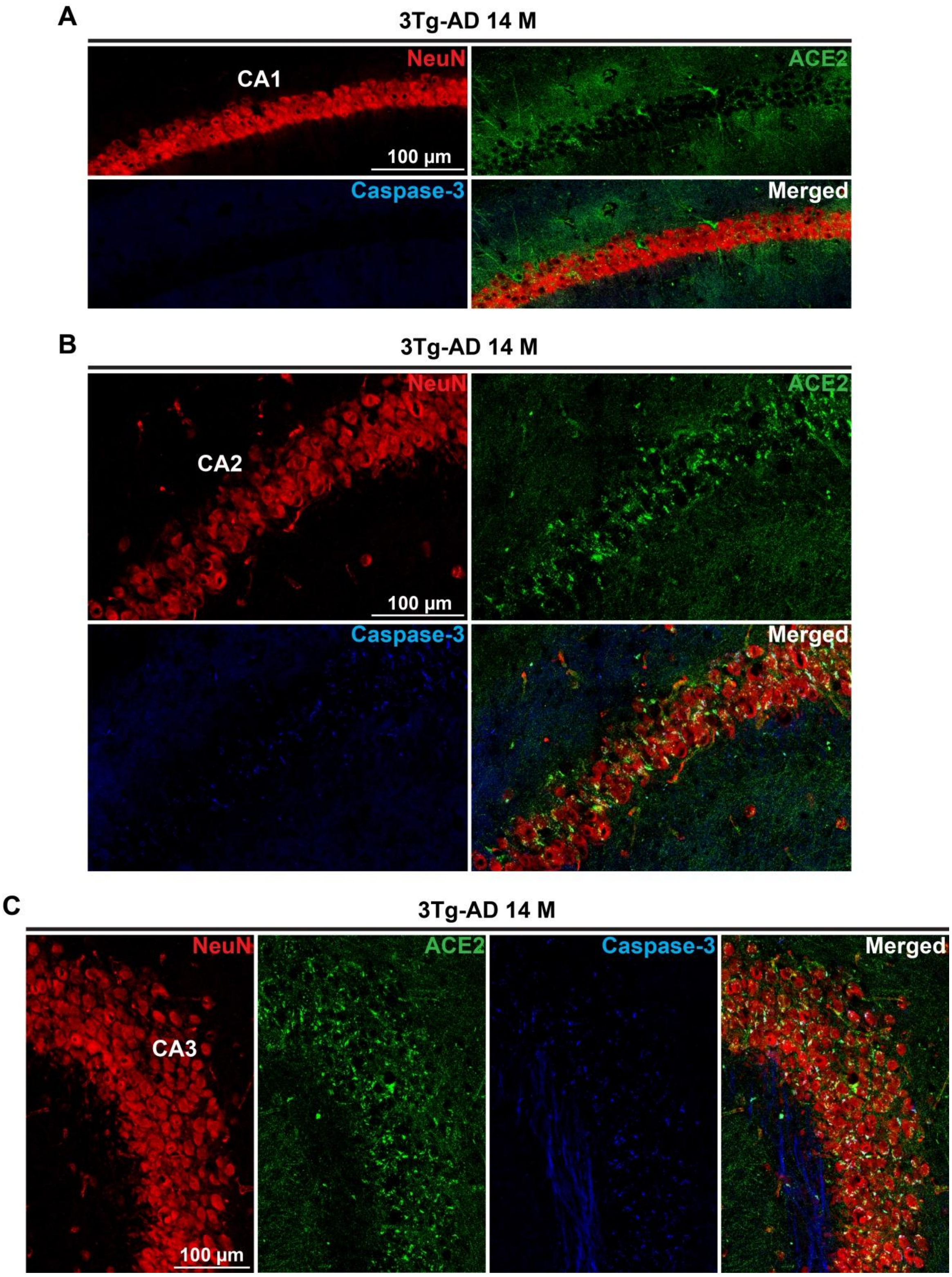

Fig. S8. ACE2 and cleaved caspase 3 staining in 3xTg-AD mice. (**A**), Immunofluorescence images of CA1. (**B**), Immunofluorescence images of CA2. (**C**), Immunofluorescence images of CA3. Scale bar = 100 μm.

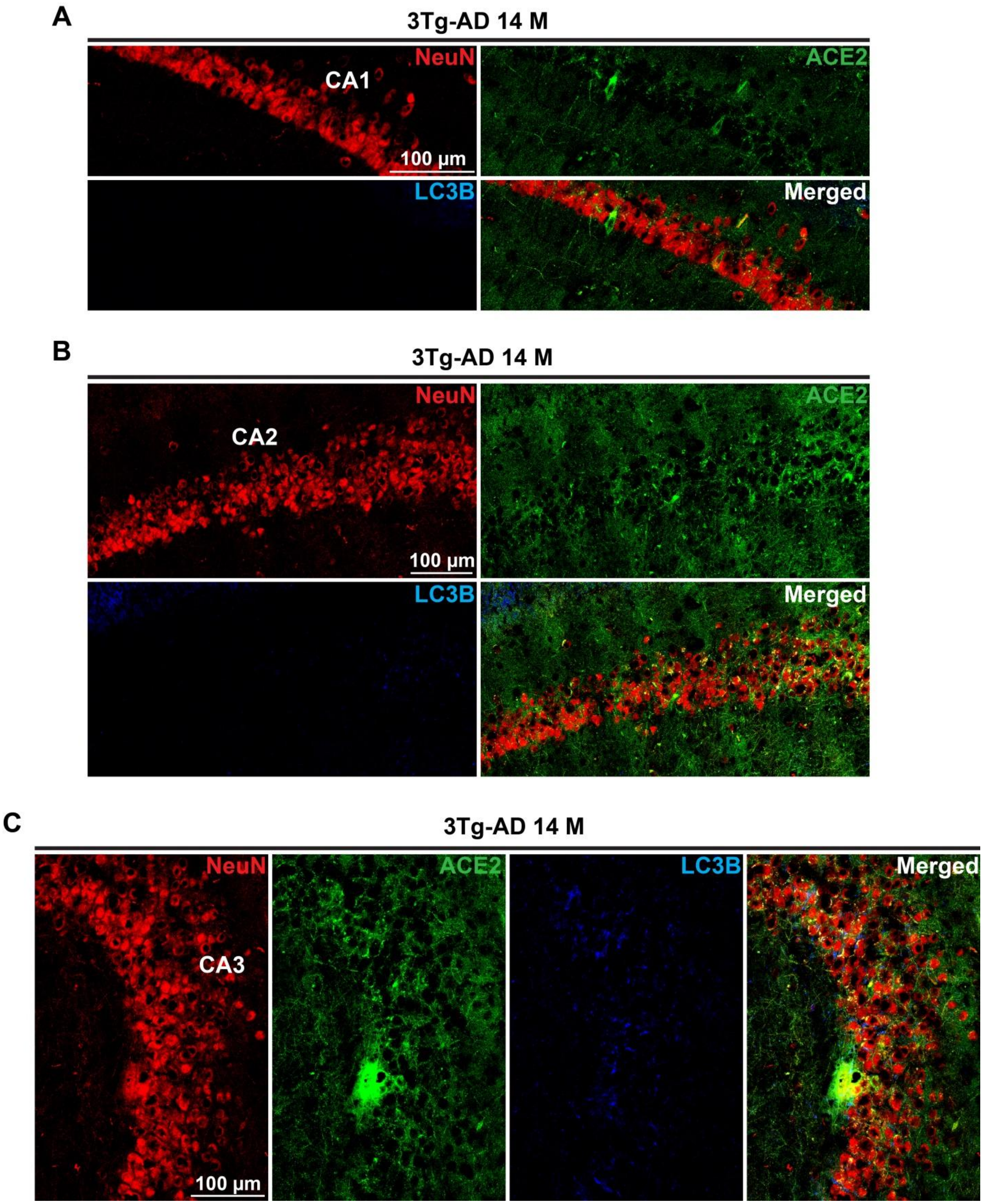

Fig. S9. ACE2 and LC3B staining in 3xTg-AD mice. (**A**), Immunofluorescence images of CA1. (**B**), Immunofluorescence images of CA2. (**C**), Immunofluorescence images of CA3. Scale bar = 100 μm.

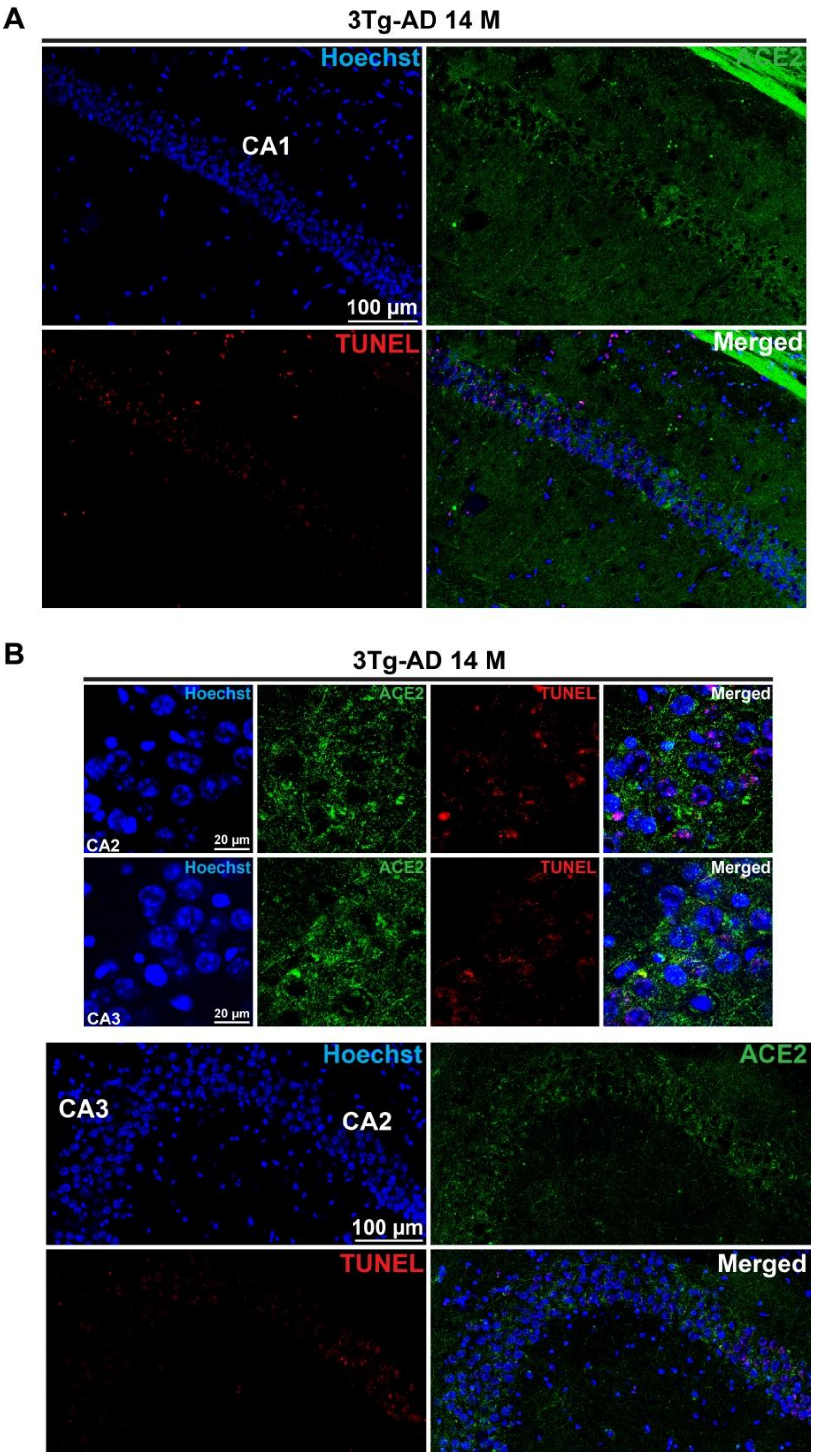

Fig. S10. ACE2 and TUNEL staining in 3xTg-AD mice. (**A**), Immunofluorescence images of CA1. Scale bar = 100 μm. (**B**), Immunofluorescence images of CA2 and CA3. Upper panel: images are in high magnifications (scale bar = 20 μm). Lower panel: images are in low magnifications (scale bar = 100 μm).

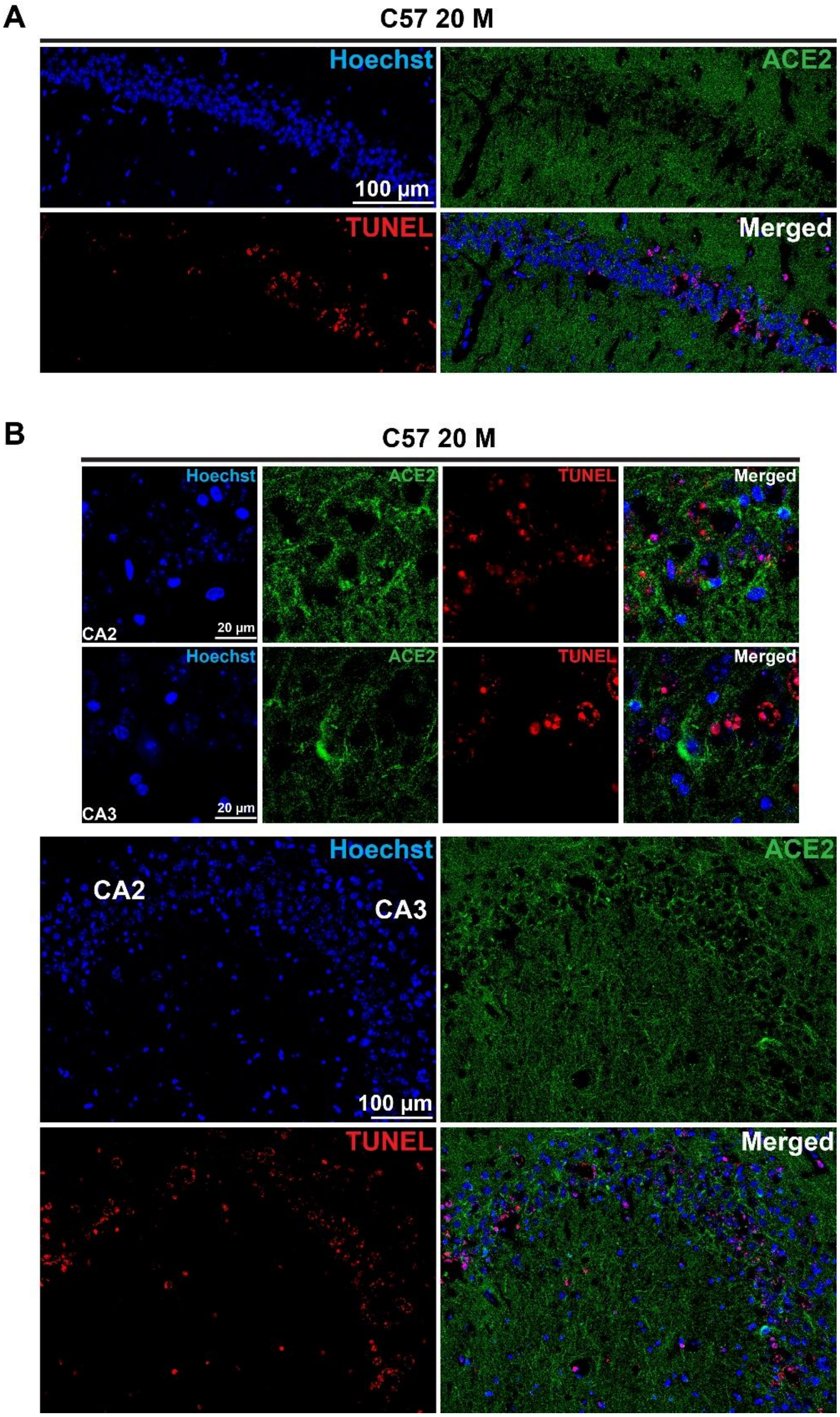

Fig. S11. ACE2 and TUNEL staining in old C57BL/6J mice. (**A**), Immunofluorescence images of CA1. Scale bar = 100 μm. (**B**), Immunofluorescence images of CA2 and CA3. Upper panel: images are in high magnifications (scale bar = 20 μm). Lower panel: images are in low magnifications (scale bar = 100 μm).

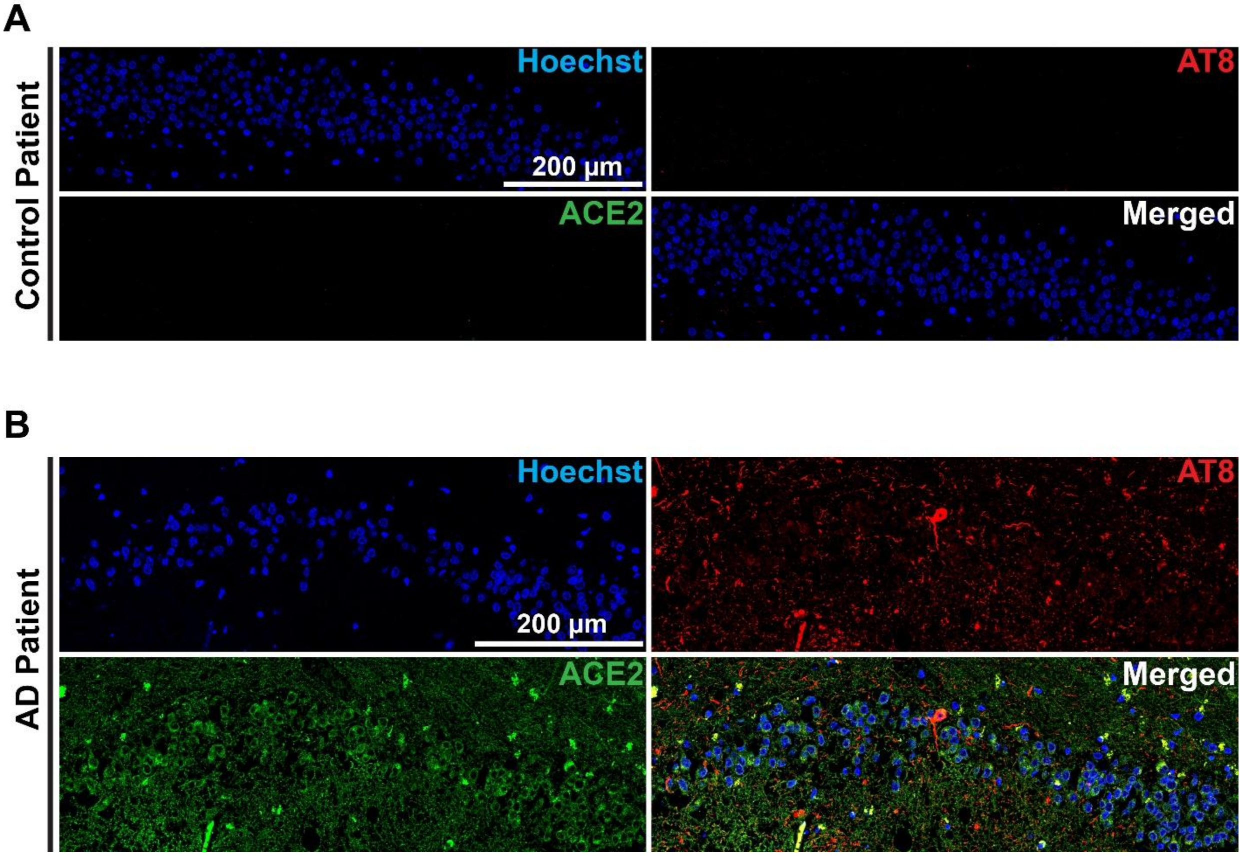

Fig. S12. ACE2 and phospho-tau staining in human hippocampus. (**A**), Immunofluorescence images of normal brain. Scale bar = 200 µm. (**B**), Immunofluorescence images of brain with AD neuropathology. Scale bar = 200 µm. Hyperphospho-tau at ser 202 and thr 205 is detected by the AT8 antibody

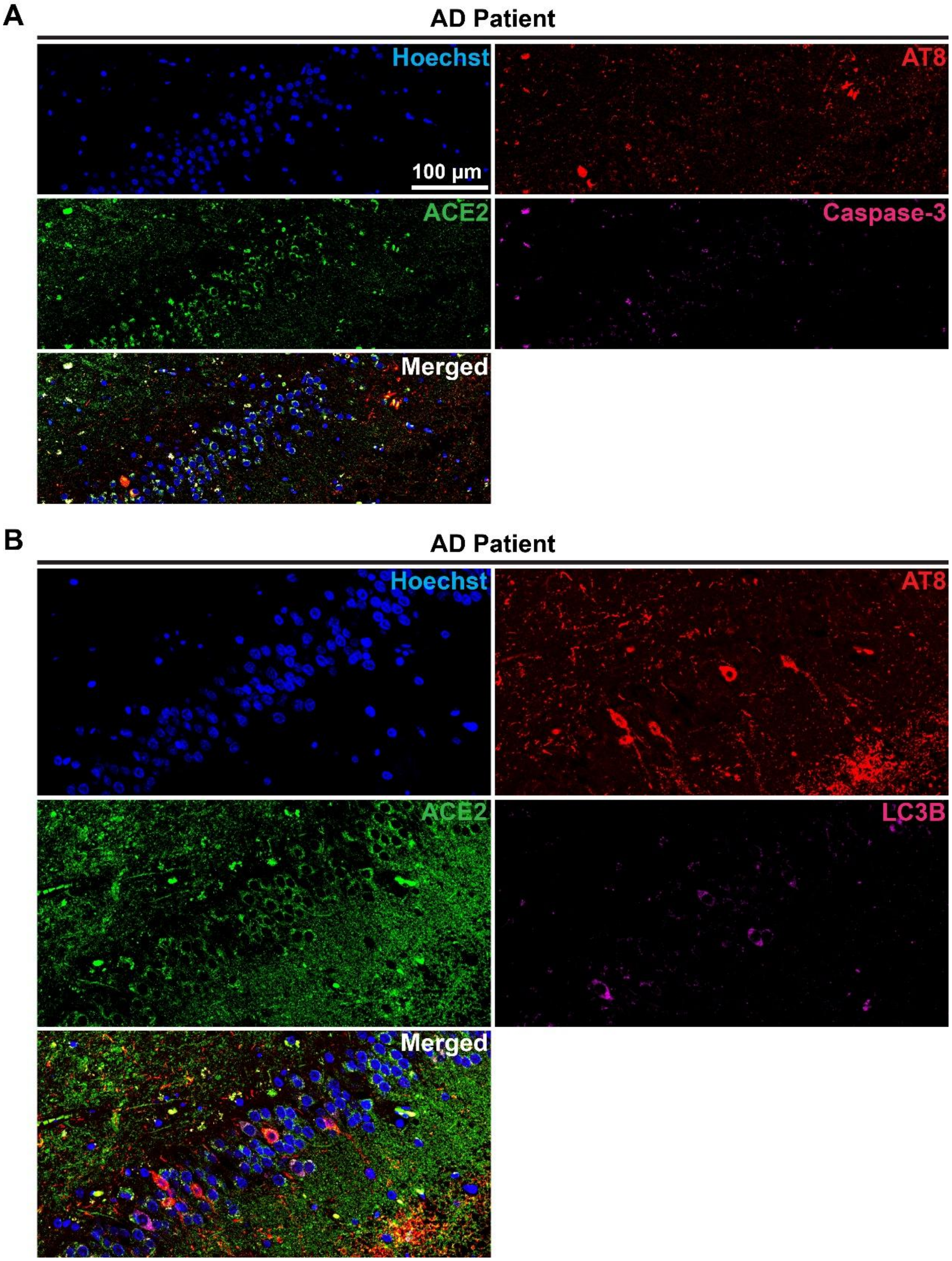

Fig. S13. ACE2, phospho-tau, cleaved caspase 3 and LC3B staining in human hippocampus. (**A**), Immunofluorescence images of triple staining of ACE2, phospho-tau and cleaved caspase 3 in brain with AD neuropathology. Scale bar = 100 μm. (**B**), Immunofluorescence images of triple staining of ACE2, phospho-tau and LC3B in brain with AD neuropathology. Scale bar = 100 μm.
